# Retraction: Activation of endoplasmic reticulum stress promotes autophagy and apoptosis and reverses chemoresistance of human small cell lung cancer cells by inhibiting the PI3K/AKT/mTOR signaling pathway

**DOI:** 10.18632/oncotarget.27064

**Published:** 2019-06-25

**Authors:** Xin-Shuang Yu, Juan Du, Yu-Jun Fan, Feng-Jun Liu, Li-Li Cao, Ning Liang, De-Guo Xu, Jian-Dong Zhang

**Affiliations:** ^1^ Department of Radiation Oncology, Shandong Provincial Qianfoshan Hospital, Shandong University, Jinan 250014, P.R. China; ^2^ Medical Research Center, Shandong Provincial Qianfoshan Hospital, Shandong University, Jinan 250014, P.R. China; ^3^ Medical Management Service Center of Shandong Provincial Health and Family Planning Commission, Jinan 250014, P.R. China

In August 2018 an investigation of this article was requested by the Supervisory Committee of the National Natural Science Foundation of China, after allegations of fraud. The Oncotarget editorial staff also requested that Shandong University bring the paper to the attention of an ethical committee in August 2018, after the corresponding authors requested retraction on the grounds of statistical errors. The institution’s investigation determined that “all the experiments of this paper were entrusted by the first author Yu Xinshuang to a commercial company, and the original experimental data were unable to be provided. Corresponding authors and other authors were unaware of the relevant experiments nor the submission process of the paper.”

Based on these results, all authors have agreed to the retraction of this article from Oncotarget.

Original article: Oncotarget. 2016; 7:76827–76839. 76827-76839 . https://doi.org/10.18632/oncotarget.12718

